# Aberrant glycosylation in schizophrenia: insights into pathophysiological mechanisms and therapeutic potentials

**DOI:** 10.3389/fphar.2024.1457811

**Published:** 2024-09-02

**Authors:** Yanchen Feng, Lu Sun, Xue Dang, Diyan Liu, Ziyun Liao, Jianping Yao, Yunke Zhang, Ziqi Deng, Jinyao Li, Min Zhao, Feixiang Liu

**Affiliations:** ^1^ The First Clinical Medical School, Henan University of Chinese Medicine, Zhengzhou, China; ^2^ Traditional Chinese Medicine (Zhong Jing) School, Henan University of Chinese Medicine, Zhengzhou, China; ^3^ College of Acupuncture, Moxibustion and Tuina, Henan University of Chinese Medicine, Zhengzhou, China; ^4^ School of Rehabilitation Medicine, Henan University of Chinese Medicine, Zhengzhou, China; ^5^ School of Pharmacy, Henan University of Chinese Medicine, Zhengzhou, China; ^6^ Hospital of Encephalopathy, The First Affiliated Hospital of Henan University of Chinese Medicine, Zhengzhou, China

**Keywords:** schizophrenia, glycosylation, proteomics, post-translational modifications, antipsychotic drugs

## Abstract

Schizophrenia (SCZ) is a severe neuropsychiatric disorder characterized by cognitive, affective, and social dysfunction, resulting in hallucinations, delusions, emotional blunting, and disordered thinking. In recent years, proteomics has been increasingly influential in SCZ research. Glycosylation, a key post-translational modification, can alter neuronal stability and normal signaling in the nervous system by affecting protein folding, stability, and cellular signaling. Recent research evidence suggests that abnormal glycosylation patterns exist in different brain regions in autopsy samples from SCZ patients, and that there are significant differences in various glycosylation modification types and glycosylation modifying enzymes. Therefore, this review explores the mechanisms of aberrant modifications of N-glycosylation, O-glycosylation, glycosyltransferases, and polysialic acid in the brains of SCZ patients, emphasizing their roles in neurotransmitter receptor function, synaptic plasticity, and neural adhesion. Additionally, the effects of antipsychotic drugs on glycosylation processes and the potential for glycosylation-targeted therapies are discussed. By integrating these findings, this review aims to provide a comprehensive perspective to further understand the role of aberrant glycosylation modifications in the pathophysiology of SCZ.

## 1 Introduction

Schizophrenia (SCZ) is a heterogeneous syndrome that severely affects the functioning of the nervous system, and its etiology involves a complex interplay of neurobiology, neurochemistry, and genetics (Birnbaum and Weinberger, 2024; Howes et al., 2024a; Howes et al., 2024b). Typical manifestations of the disease include impaired cognitive functioning, emotional abnormalities, and social dysfunction (Sun, 2023). Patients often experience severe symptoms such as hallucinations, delusions, emotional apathy, and disorganized thinking (Suárez Santiago et al., 2023; Humpston and Woodward, 2024). Due to its chronic degenerative nature, prolonged course, recurrent episodes, and high disability rate, SCZ has become one of the most significant mental health burdens globally, bringing great psychological, social, and economic burdens to patients, their families, and society (Wander, 2020; Modi et al., 2021). According to the Global Burden of Disease data for 205 countries and territories from 1990 to 2019, the prevalence of SCZ is approximately 0.3%–0.7%, based on the diagnostic criteria of the fourth edition of the Diagnostic and Statistical Manual of Mental Disorders and the 10th edition of the International Classification of Diseases. The onset of the disease spans a wide age range but is most observed in adolescents and young adults, particularly those between the ages of 20 and 30 years (Collaborators, 2022). Currently, the treatment of SCZ is primarily based on a multifaceted and comprehensive strategy that aims to alleviate symptoms, enhance patients’ quality of life, and reduce relapse rates. Common first-line treatments include first-generation antipsychotics such as chlorpromazine and sulpiride, as well as second- and third-generation medications such as clozapine, risperidone, aripiprazole, and lurasidone, which are less likely to cause extrapyramidal reactions (most common extrapyramidal adverse effects include acute dystonia, akathisia, parkinsonism, and tardive dyskinesia) (Leucht et al., 2023; Szmulewicz et al., 2024). In addition, psychotherapeutic approaches such as cognitive-behavioral therapy and family therapy are also important components of treatment (O'Driscoll et al., 2014; Daruvala et al., 2021). However, since the etiology of SCZ is still unclear, early diagnosis is not only difficult but also prone to missed and incorrect diagnoses in clinical practice. In addition, long-term use of antipsychotics may lead to the development of side effects such as movement disorders (e.g., tardive dyskinesia and parkinsonism), metabolic disturbances (e.g., weight gain, dyslipidemia, and insulin resistance), cardiovascular disorders (e.g., hypertension and cardiac arrhythmias), hyperprolactinemia, and cognitive dysfunction (Haddad and Wieck, 2004; Haupt, 2006; Bostwick et al., 2009; Pillinger et al., 2020; Schneider-Thoma et al., 2021). Therefore, a deeper understanding of the pathophysiology of SCZ is crucial for improving the diagnosis and treatment of SCZ in clinical settings, as well as for enhancing patients’ prognoses.

Genetic predisposition and environmental factors are considered the main risk factors for triggering SCZ, but proteomics-based research on SCZ is a prominent research direction (Shen et al., 2024). Through proteomics technology, determining the changes in protein expression levels and related biochemical pathways in tissue samples from SCZ patients can further deepen our understanding of the biochemical mechanisms of SCZ (Luo et al., 2024). Protein post-translational modifications (PTMs) is a crucial part of proteomics research. After ribosomal synthesis, proteins undergo chemical modifications to extend their functions beyond their basic sequences, enabling them to respond dynamically to cellular and environmental signals (Csizmok and Forman-Kay, 2018). PTMs can alter the chemical properties, spatial conformation, and interaction ability of proteins, which in turn affects the normal function of cells and organisms. Notably, PTMs play key roles throughout the entire life cycle from protein synthesis to degradation and have a decisive impact on the diversity of protein functions and the regulation of their key attributes (e.g., enzyme activity, interaction potential, intracellular localization, and overall stability). PTMs come in a variety of forms including, but not limited to, phosphorylation, glycosylation, ubiquitylation, acetylation, methylation, and sulfation (Didonna and Benetti, 2016). These modifications can occur in various environments and on specific residues of proteins, providing cells with a versatile toolkit to finely regulate protein function.

Glycosylation, a process in which sugar molecules are covalently bound to proteins and lipids through enzymatic reactions, plays an important role in regulating physiological and pathological states (Reily et al., 2019). As a key biochemical regulatory mechanism, it involves nearly all cellular pathways and disease states in eukaryotic organisms. Glycosylation can be categorized into four main types: N-glycosylation, O-glycosylation, C-glycosylation, and GPI-anchored glycosylation, with N- and O-glycosylation being more intensively studied. N-glycosylation is the process by which a sugar group is linked to an asparagine (Asn) residue in a protein via a nitrogen atom. This process typically occurs in the endoplasmic reticulum and the Golgi apparatus (Pongracz et al., 2024). O-glycosylation is the process by which a sugar group is linked to a serine (Ser) or threonine (Thr) residue in a protein via an oxygen atom. This process usually occurs in the Golgi apparatus (Li et al., 2022). It has been reported that more than 70% of brain proteins undergo glycosylation modifications (Zhang et al., 2024). Glycoproteins and glycolipids on the surface of neural stem cells have been shown to play key roles in biological processes such as neural development, cell adhesion, receptor activation, signal transduction, neuronal differentiation and migration, synapse extension, and synaptogenesis (Iqbal et al., 2019; Kimura et al., 2021). These complex glycosylation processes are not only crucial for normal brain function but also closely related to the onset and development of various neurological diseases. As early as 1980, researchers identified significant abnormalities in the serum glycoproteins of SCZ patients through a comparative study. Serum samples from 30 SCZ patients were compared with those from 20 healthy controls. The results demonstrated that the protein-bound carbohydrate components in the serum of SCZ patients, including glucose and arabinose, were significantly elevated. Additionally, electrophoretic profiles revealed increased levels of alpha-2 and beta-globulins (Varma and Hoshino, 1980). Further studies found that spherical deposits of glycan components in the dentate gyrus of the hippocampus of SCZ patients could accelerate neuronal degeneration, leading to dysfunction of the dentate gyrus neural network, which in turn affected cognitive and memory functions (Walton et al., 2012). Genome-wide association analysis of the dorsolateral prefrontal cortex (DLPFC) in SCZ patients also revealed that the biosynthetic pathways of N-glycans and O-glycans were highly enriched (Mealer et al., 2020). Additionally, aberrant expression and mutation of multiple SCZ candidate genes also lead to altered glycosylation modifications (Mealer et al., 2020). Therefore, aberrant glycosylation modifications have been recognized as an important link in the pathomechanism of SCZ. To systematically summarize existing research and reveal the potential mechanisms of glycosylation in SCZ, this review aims to organize and assess the current status of research on the relationship between glycosylation and SCZ, identify key issues and gaps in the research, and propose future research directions (Table 1).

**TABLE 1:** Abnormal glycosylation modifications in SCZ.

	Tissue		Sample size			Changes in glycosylation	References
	Category	Type	N	Individuals with SCZ	Healthy controls		
N-glycosylation	Brain (Autopsy)	DLPFC	67	35	31	GluA2 subunit (Man↓)	Tucholski et al. (2013a)
	Brain (Autopsy)	DLPFC	67	35	31	GluK2 subunit (Man↑) (Hybridization↓)	Tucholski et al. (2013b)
	Blood	Serum	51	17	34	GluK2/3 (N-glycosylation↓)	Ambalavanan et al. (2016)
	Brain (Autopsy)	Anterior cingulate cortex; DLPFC	68	35	33	EAAT1 ↓ EAAT2 ↓	Bauer et al. (2010)
	Brain (Autopsy)	STG	28	14	14	GABA_A_R *β*2 subunit (N-glycosylation↑)	Mueller et al. (2014)
	Cell	STG isolates subcellular	30	16	14	GABA_A_R *β*2 subunit↑	Mueller et al. (2015)
O-glycosylation	Brain (Autopsy)	STG	20	10	10	GALNT16↓	Mueller et al. (2019a)
	Brain (Autopsy)	STG	34	17	17	O-GalNaAc↓; O-GlcNAcylation↑	Mueller et al. (2018a)
Glycosyltransferases	Brain (Autopsy)	DLPFC	24	12	12	UGGT2↑	Kim et al. (2018)
	Brain (Autopsy)	DLPFC	24	12	12	B3GNT8↓; MGAT4A↓	Kippe et al. (2015)
	Brain (Autopsy)	STG	30	16	14	POFUT2↑; FUT8↓	Mueller et al. (2017)
PSA-NCAM	Brain (Autopsy)	Hippocampus	36	21	21	PSA↓; Protein adhesive↓	Vawter et al. (2001)
	Blood	Serum	106	55	51	PSA↑; Hippocampal gray matter volume↓	Müller-Miny et al. (2023)
	Blood; Brain	Serum; Hippocampus	88	44	44	PSA-NCAM↑ brain integrity↓	An et al. (2020)
	ST8SIA2 knockout mice	Hippocampus	Unknow	8	Unknow	ST8SIA2↓	Kröcher et al. (2015)
	Brain (Autopsy)	STG	30	16	14	ST8SIA2↑	Mueller et al. (2018b)

## 2 N-glycosylation in SCZ

Glycosylation takes place in the endoplasmic reticulum (ER), where high-mannose (Man) “precursors” are attached to the target protein by means of a process known as integral transfer (Hirata and Kizuka, 2021). This intricate process involves the coordinated action of multiple enzymes, each executing a specific step within a unified pathway to ensure that sugar molecules are correctly attached to proteins, lipids, or other molecules (Bieberich, 2014). At the ER membrane, dolichol phosphate acts as a glycosyl donor, facilitating the synthesis of a glycan precursor composed of 14 sugar molecules. These molecules consist of 2 N-acetylglucosamine (GlcNAc), 9 Man, and 3 glucose (Glc) units (Schallus et al., 2008; Schoberer et al., 2024). Once the glycan precursor synthesis is complete at the ER membrane, the precursors are transferred to Asn residues on the nascent polypeptide chain by glycosyltransferases. This typically occurs in the specific sequence motif Asn followed by any amino acid except proline, and then serine or threonine (Asn-Any Amino Acid-Ser/Thr) (Hirata and Kizuka, 2021). Following the glycosyl transfer, the initial N-glycan chain (characterized by a high Man content) undergoes trimming of Glc and some Man residues by glycosidases within the ER (Frappaolo et al., 2020). Subsequently, glycosyltransferases in the Golgi apparatus further modify the glycan by adding monosaccharides such as GlcNAc, galactose (Gal), fucose (Fuc), and sialic acid (Sia), resulting in a diverse array of heterotrimeric and complex N-glycosyl chains (Nagae et al., 2020; Oka et al., 2022).

**FIGURE 1:**
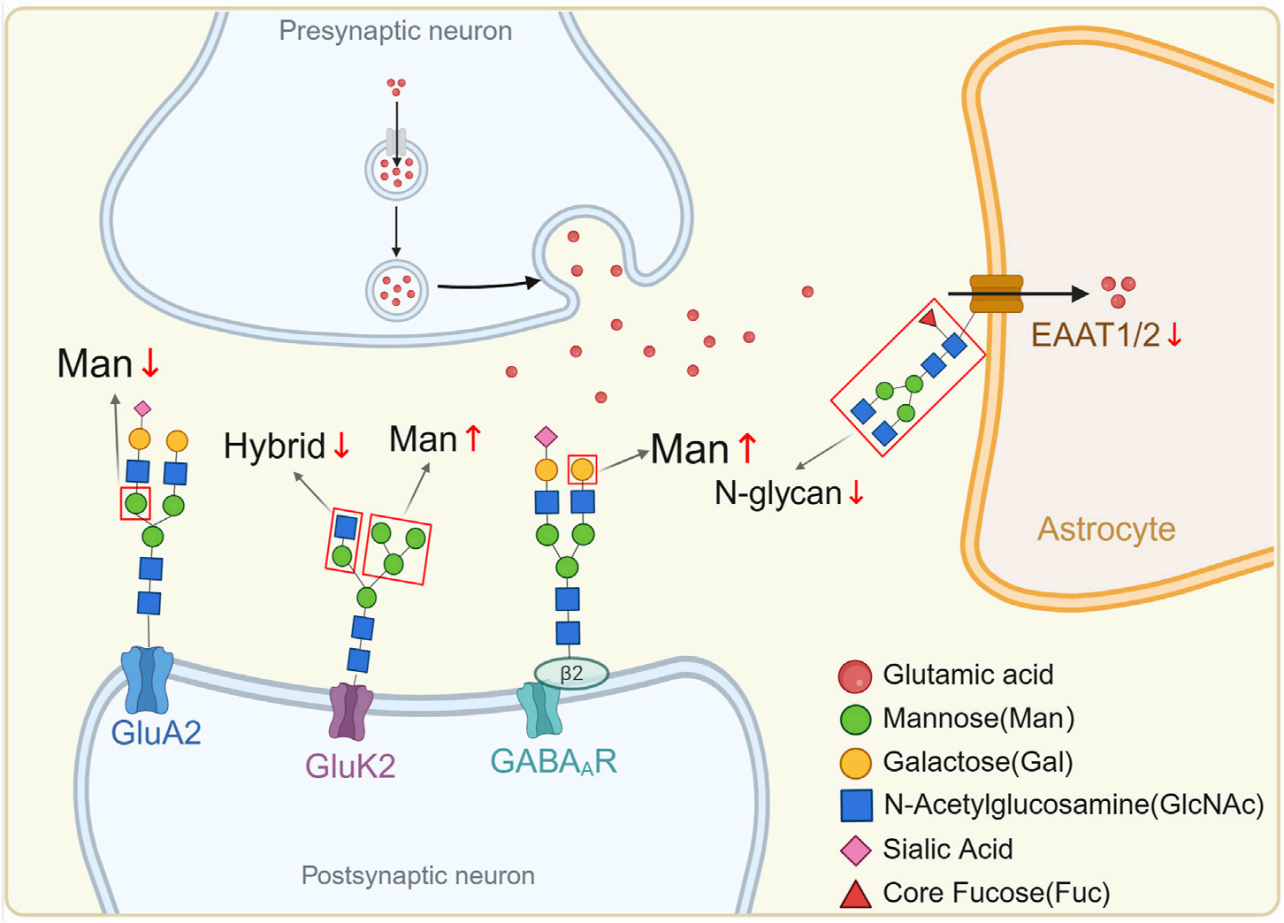
Abnormal N-glycosylation in SCZ.

As one of the most abundant PTM, N-glycosylation plays a crucial role in proper protein folding, stability, intercellular interactions, and signaling (Price et al., 2010). N-glycosylation modifications exhibit species- and tissue-specificity and undergo remodeling in both type and content during brain development and aging (Barboza et al., 2021). These modifications are integral to the regulation of neuronal activity, synaptic transmission, and the release and recycling of neurotransmitters. A study of N-glycan abundance in the mouse and human prefrontal cortex (PFC) identified 136 identical N-glycans and 6 different N-glycans between the two species, suggesting high compatibility in N-glycosylation between humans and mice (Lee et al., 2020). Furthermore, N-glycans in the mouse brain exhibited spatial specificity, showing significant differences in glycan abundance across nine brain regions (Noel et al., 2023). In contrast, 36 glycans demonstrated time-specific expression in samples from the PFC of healthy human tissue (Lee et al., 2020). This dynamic expression profile of N-glycosylation modifications suggests their critical functions in neurodevelopment and the maintenance of neurological homeostasis. Additionally, N-glycosylation enhances the water solubility of proteins, prevents their aggregation, and plays an essential role in cell surface recognition and immune responses (Zhou and Qiu, 2019). It has been shown that the accumulation of insoluble protein aggregates is significantly associated with the progression of neurodegenerative diseases (Gozal et al., 2009). Consequently, dysfunction in key glycosylation enzymes can cause disruptions in the N-glycosylation pathway, leading to diseases such as congenital disorders of glycosylation (CDGs), which present with severe clinical symptoms (Jaeken, 2011). Notably, nearly all CDGs are characterized by neurologically relevant abnormalities, such as cerebral dysplasia, epilepsy, and intellectual disability (Drouin-Garraud et al., 2001; Fiumara et al., 2016). Thus, N-glycosylation modifications are crucial for the development and function of the nervous system, and abnormalities in these modifications may contribute to the pathophysiological changes seen in SCZ, a complex neuropsychiatric disorder. Although the etiology of SCZ is not yet fully understood, growing evidence suggests that N-glycosylation modifications play a significant role (Figure 1).

In the presynaptic neuron, neurotransmitters such as glutamic acid are released into the synaptic cleft. The postsynaptic neuron contains receptors such as GluA2, GluK2, and GABA_A_, which exhibit abnormal glycosylation patterns in SCZ. Specifically, there is a decrease in mannose (Man↓) on GluA2, a decrease in hybrid glycan structures (Hybrid↓) on GluK2, and an increase in Man (Man↑) on GABA_A_ receptors. Additionally, the EAAT1/2 transporter also exhibited decreased levels of N-glycosylation (N-glycan↓). These glycosylation changes, highlighted by the abnormalities in expression levels of various glycans such as Man, Gal, GlcNAc, Sia, and core Fuc, contribute to the altered function and regulation of synaptic receptors and transporters, thereby implicating them in the pathophysiology of SCZ. All glycan structures in the figure are based on the fourth edition of Essentials of Glycobiology (https://www.ncbi.nlm.nih.gov/glycans/snfg.html).

### 2.1 The glutamate hypothesis in SCZ and N-glycosylation

Glutamate is the primary excitatory neurotransmitter in the central nervous system (Shank and Aprison, 2018). It plays a crucial role in learning, memory, and neuroplasticity through glutamate receptor-mediated synaptic transmission, including N-methyl-D-aspartate (NMDA) receptors, *α*-amino-3-hydroxy-5-methyl-4-isoxazolepropionic acid (AMPA) receptors, and kainate (KA) receptors (Flores-Soto et al., 2013; Hansen et al., 2021). These receptors each have unique transport and localization mechanisms that ensure their proper function at the right time and place. Previous studies have shown that the transport of glutamate receptors is disrupted in patients with SCZ (Marsman et al., 2013). Since N-glycosylation modification is closely related to the process of protein synthesis and final localization, and plays a crucial role in protein folding, stability, transport, and function, the abnormal modification of N-glycosylation in patients with SCZ directly reflects the pathological state of abnormal glutamate receptor transport.

#### 2.1.1 Abnormal N-glycosylation of AMPA receptors

AMPA receptors are one of the main types of glutamate receptors mediating fast excitatory synaptic transmission. They are widely distributed throughout the central nervous system and consist of four subunits (GluA1, GluA2, GluA3, GluA4). Each subunit contains an N-terminal extracellular domain, a ligand-binding domain (LBD), a transmembrane domain (TMD), and a C-terminal intracellular domain (Cao et al., 2023; Corti and Duarte, 2023). AMPA receptors mediate fast synaptic transmission and produce an excitatory postsynaptic potential by opening ion channels through a conformational change triggered by glutamate binding to the LBD, allowing the influx of sodium and calcium ions (Zhang et al., 2008). AMPA receptors play a crucial role in synaptic plasticity, regulating the efficiency of synaptic transmission through long-term potentiation (LTP) and long-term depression (LTD), which affect learning and memory processes (Diering and Huganir, 2018). During LTP, the number of receptors increases, while during LTD, the number of receptors decreases, achieved through insertion and endocytosis mechanisms, respectively (Díaz-Alonso and Nicoll, 2021).

The function and localization of AMPA receptors are regulated by multiple PTMs, including phosphorylation, glycosylation, and ubiquitination (Corti and Duarte, 2023). Among these, AMPA receptors undergo complex N-glycosylation modifications that affect their folding and transport (Tucholski et al., 2014). Enzyme-catalyzed deglycosylation studies of DLPFC samples from patients with SCZ revealed that both GluA2 and GluA4 undergo N-glycosylation modifications, and the proportion of GluA2 containing high Man was significantly reduced (Tucholski et al., 2013a). This phenomenon is related to the editing of glutamic acid (Glu607) to arginine (Arg607) in the TMD of the GluA2 subunit (Greger et al., 2002). The edited GluA2 is primarily retained in the ER, whereas the unedited GluA2 is more easily transported to the synapse. In SCZ, the proportion of unedited GluA2 is significantly increased, resulting in its faster departure from the ER and transport to the synapse, thereby affecting synaptic function. Additionally, the increased insensitivity of GluA2 to Endo H (a de-glycosylase) and the decreased reactivity to ConA (a lectin that recognizes Man) indicate that the extension and maturation of the glycan chain are compromised. In summary, the abnormal processing and transport of GluA2 during glycosylation may be a key pathological mechanism underlying the imbalance of neurotransmission and cognitive dysfunction in SCZ.

#### 2.1.2 Abnormal N-glycosylation of the KA receptor

The KA receptor is an ion channel composed of five distinct subunits (GluK1-5), each assembled into a tetramer from four subunits (either homo- or heterodimeric) (Falcón-Moya and Rodríguez-Moreno, 2021). These subunits are classified into two subfamilies based on their sequence and function: GluK1-3 (low-affinity receptors) and GluK4-5 (high-affinity receptors) (Watanabe-Iida et al., 2016). KAs play various roles in the central nervous system, primarily categorized into presynaptic and postsynaptic effects (Negrete-Díaz et al., 2022). Presynaptically, KAs can modulate neurotransmitter release by regulating calcium ion permeability and inhibiting voltage-dependent potassium channels upon activation (Sakha et al., 2016). The modulation pattern depends on the combination of different KA subunits. Additionally, KAs affect peripheral neurons by regulating the release of their neurotransmitters (e.g., glutamate) and paracrine neurotransmitters (e.g., GABA) (Kullmann, 2001). Postsynaptically, KAs facilitate the influx of sodium and calcium ions, leading to depolarization and the transmission of excitatory signals, thus regulating synaptic strength and influencing learning and memory processes (Malenka et al., 1988). A study on samples of the DLPFC from patients with SCZ treated with glycosidase revealed that the N-glycosylation of GluK2 was more sensitive to de-glycosylase (Endo H) (Tucholski et al., 2013b). This finding suggests that in SCZ patients, GluK2 has more high-Man structures and fewer hybrid structures, causing it to remain in the ER or Golgi apparatus longer, thus inhibiting normal transport and limiting its assembly into functional KAs. Impaired KA receptors consequently damage pre- and postsynaptic neurotransmission and neuroplasticity in SCZ patients, affecting cognitive function and behavioral performance (Negrete-Díaz et al., 2022).

Human natural killer cell-1 (HNK-1) is a specific glycosylation modification that is completed in the trans-Golgi network. It contains a trisaccharide structure composed of sulfated glucuronic acid, Gal, and GlcNAc (Yabuno et al., 2015). Seizure-related gene 6 (SEZ6) promotes the transport of GluK2 and GluK3 to the ER and interacts with these subunits, ensuring their proper transport to the Golgi for HNK-1 modification (Pigoni et al., 2020). This process maintains their glycosylation and correct surface localization, thereby playing a key role in the regulation of synaptic connections and LTP (Nair et al., 2023). Additionally, the transmembrane protein SEZ6 is crucial for regulating the activity of KA receptors by promoting the transport of GluK2 and GluK3 subunits to the cell surface, thus influencing the glycosylation status of the neuronal surface (Pigoni et al., 2020). When SEZ6 is knocked down in primary neurons, the levels of GluK2 and GluK3 on the cell surface are significantly reduced (Gurung et al., 2018). In the regulatory mechanism involving SEZ6 and HNK-1, the transferase *β*-1,3-glucuronyltransferase 1 (B3GAT1), which participates in the glycosylation process, can produce a positional effect, regulate LTP and spatial memory formation in the mouse hippocampus, and participate in the synthesis of HNK-1 antigens (Jeffries et al., 2003). B3GAT1 has also been identified as a key candidate gene for SCZ (Jeffries et al., 2003). Exome sequencing of patients with childhood-onset SCZ revealed a deletion of the extracellular structural domain of the SEZ6 gene. The deletion of this structural domain prevents the glycosylation modification of GluK2/3 and affects the modification process of HNK-1, which mediates neuronal function and connectivity and regulates early brain development (Ambalavanan et al., 2016). Overall, these studies provide evidence for abnormal glycosylation of the KA receptor subunit in SCZ, further elucidating the potential mechanisms and roles of GluK2 and GluK3 in SCZ.

#### 2.1.3 Abnormal N-glycosylation of amino acid transporters

Excitatory amino acid transporters (EAATs) are membrane transport proteins responsible for glutamate uptake, primarily found in neurons and glial cells of the central nervous system (Malik and Willnow, 2019). Among these, EAAT1 and EAAT2 are the main glutamate transporters and are also N-glycosylated proteins. They clear glutamate from the synaptic cleft, regulate its concentration, and maintain normal neurotransmission (Parkin et al., 2018). EAAT1 is highly expressed in the cerebellum and produces 64 kDa and 70 kDa glycoproteins through glycosylation, which enhances protein stability, membrane localization, and function (Conradt et al., 1995). Similarly, EAAT2, expressed mainly in the cerebral cortex and hippocampus, produces a 5–15 kDa glycoprotein via glycosylation. N-glycosylation of EAAT2 is crucial for its stability, membrane localization, and transport function (Takahashi et al., 2015). Deglycosylation of EAAT2 leads to insufficient glutamate clearance, elevated synaptic glutamate levels, and neurotoxicity due to NMDA receptor overactivation or inactivation, causing synaptic dysfunction. Animal studies have shown that EAAT1 or EAAT2 knockout mice exhibit SCZ-like behavioral and cognitive abnormalities (Karlsson et al., 2009). Human autopsy studies of the anterior cingulate cortex and DLPFC indicate reduced N-glycosylation levels of EAAT1 and EAAT2 in SCZ patients (Bauer et al., 2010). This reduction decreases the molecular mass shift, inhibiting the expression of these transporters on the plasma membrane and impairing glutamate reuptake. These findings suggest that EAAT1 and EAAT2 proteins in SCZ exhibit accelerated exit from the ER and abnormal forward transport.

As previously discussed, the N-glycosylation modifications of AMPA receptors and KA receptors exhibit contrasting mechanisms in patients with SCZ. Specifically, N-glycosylation enhances the forward transport and functional activity of AMPA receptors, whereas it causes retention and functional impairment of KA receptors within the ER or Golgi apparatus. Additionally, decreased N-glycosylation levels of EAAT1 and EAAT2 transporters disrupt glutamate uptake. These findings underscore the significant role of N-glycosylation in the pathophysiology of SCZ and emphasize the consequences of aberrant N-glycosylation on the processing, trafficking, and neurotransmitter release of glutamate receptors and transporters.

### 2.2 The GABA hypothesis in SCZ and N-glycosylation

Gamma-aminobutyric acid (GABA) is the primary inhibitory neurotransmitter in the central nervous system (Yang and Tsai, 2017; Maramai et al., 2020). It regulates inhibitory synaptic transmission through its receptors, such as GABA_A_ and GABA_B_ receptors, maintaining the neural network balance and preventing neurotoxicity from excessive excitatory transmission. The GABA hypothesis suggests that one of the pathological features of SCZ patients is the abnormal function of the GABA system, including changes in GABA synthesis, release, uptake, and receptor function, which lead to cognitive deficits (Schmidt and Mirnics, 2015). The GABA_A_ receptor is an ion channel receptor composed of various subunits (such as *α*, *β*, *γ*, *δ*, *ε*, etc.) and is a heteropentameric glycoprotein, usually consisting of 2*α* subunits, 2*β* subunits, and 1*γ* subunit (Ghit et al., 2021). This receptor induces the opening of a chloride ion channel, hyperpolarizing the neuron’s membrane potential and producing an inhibitory postsynaptic potential. Its rapid response makes it the main inhibitory receptor in the nervous system, regulating neuronal excitability and synaptic plasticity (Brady et al., 2018). The GABA_B_ receptor is a G protein-coupled receptor composed of GABA_B_1 and GABA_B_2 subunits. Unlike GABA_A_ receptors, GABA_B_ receptors regulate potassium and calcium channels by activating second messenger systems (such as inhibiting adenylate cyclase or activating phospholipase C), thereby producing a prolonged inhibitory effect. These receptors primarily regulate neurotransmitter release and long-term synaptic inhibition (Zemoura et al., 2019; Fritzius et al., 2022).

It has been reported that the density of GABA_A_ receptors in the left superior temporal gyrus (STG) of patients with SCZ is significantly increased (Deng and Huang, 2006). Concurrently, the levels of the GABA_A_ receptor *ε* subunit and *α*2 subunit proteins in the cerebellum are also significantly elevated, whereas the level of the *β*1 receptor protein is markedly decreased (Fatemi et al., 2013). Several studies have demonstrated that alterations in the level or pattern of N-glycosylation of GABA receptor subunits can influence the processing, assembly, receptor stability, and cell surface expression of mature GABA receptors, thereby impacting channel gating properties and receptor function (Cai et al., 2005; Lo et al., 2010). This suggests a potential mechanism for N-glycosylation modification in the physiology and pathology of SCZ. An enzymatic deglycosylation and lectin affinity analysis of DLPFC tissue from healthy individuals revealed N-glycosylation of the *α*1, *α*4, *β*1, *β*2, and *β*3 subunits of the GABA_A_ receptor (Mueller et al., 2014). Subsequent analysis of the DLPFC from SCZ patients showed that, compared to healthy tissue, the *α*1 subunit exhibited reduced high-Man N-glycosylation and a smaller molecular mass change post-deglycosylation. The *β*1 subunit displayed increased high-Man N-glycosylation, with a new 45 kDa subtype appearing post-deglycosylation. Additionally, the highest molecular mass subtype of the *β*2 subunit showed a significant increase after N-glycosylation (Mueller et al., 2014). These abnormal N-glycosylation changes suggest that GABA_A_ receptors in the DLPFC of SCZ patients may undergo aberrant processing and function. To substantiate this hypothesis, Mueller et al. (2015) conducted further deglycosylation of subcellular fractions of the STG and found that the expression of the *β*1 subunit in the ER fraction was relatively reduced, whereas the relative abundance of the *β*2 subunit was increased. In the synaptic fraction, *β*2 subunit expression was significantly elevated, while *β*1 subunit expression was relatively diminished.

In summary, the abnormal N-glycosylation and subcellular localization of GABA_A_ receptors in SCZ patients may lead to impaired processing, assembly, stability, and function of these receptors. Such changes could affect the channel gating properties and cell surface expression of GABA_A_ receptors, thereby disrupting GABAergic signaling and contributing to the pathophysiological changes observed in SCZ.

## 3 O-glycosylation in SCZ

O-glycosylation primarily occurs in the Golgi apparatus and involves the direct addition of monosaccharides to serine or threonine residues of proteins (Zhang and Ten Hagen, 2019). This process is typically catalyzed by N-acetylgalactosamine transferases, which add GalNAc to serine or threonine residues (Bennett et al., 2012). It represents the most common and abundant form of O-glycosylation. Once the initial GalNAc is incorporated, the sugar chain can be further extended by various glycosyltransferases, which add different monosaccharides such as Gal, Glc, Fuc, and Sia to form O-glycans (Magalhães et al., 2021). Notably, O-glycosylated sugar chains are relatively simple, ranging from monosaccharides to short-chain polysaccharides, and exhibit numerous O-glycan subtypes with distinct characteristics and functions. Unlike N-glycosylation, O-glycosylation plays a crucial role in protein secretion, ensuring that proteins are accurately transported to the extracellular space or cell membrane by regulating their stability and folding (May et al., 2020). Moreover, O-glycosylation is essential for cell adhesion, modulating cell-cell interactions and communication by altering cell surface proteins such as integrins and selectins (Tran and Ten Hagen, 2013). O-glycosylation is also vital in cell signaling, influencing signaling processes by modifying receptor proteins and ligands.

It has been reported that patients with SCZ exhibit a serum O-glycosylation product, GalNAc*α*1-3GalNAc (disialogalactosylated O-glycoprotein), which can increase the climbing behavior of mice in the forced swimming test by affecting the activity of dopamine D1 receptors (Masuda et al., 2001). This finding preliminarily indicates that O-glycosylation abnormalities may be associated with behavioral and mood disturbances. Additionally, the expression level of Polypeptide N-acetylgalactosaminyltransferase 16 (GALNT16) in the left STG tissue of SCZ patients was significantly decreased (Mueller et al., 2019a). GALNT16 is a key enzyme in the Golgi apparatus responsible for catalyzing the biosynthesis of O-GalNAc glycans. A reduction in its level may impact the formation of multiple O-GalNAc structures in the TGF-*β* signaling pathway (Herr et al., 2008). Furthermore, O-GlcNAcylation, a specific type of glycosylation step, is regulated by O-GlcNAc transferase and O-GlcNAcase, dynamically adding or removing O-GlcNAc modifications on proteins, thereby rapidly regulating protein function (Yang and Qian, 2017). Muller et al. (2018) found that the O-GalNAc level in the STG tissue of SCZ patients was significantly reduced, while the O-GlcNAcylation level was significantly increased. The decrease in O-GalNAc levels may lead to abnormal protein folding, increasing the risk of protein degradation or aggregation. Conversely, increased O-GlcNAcylation is associated with an enhanced stress response and neuronal dysfunction (Stewart et al., 2020). Further studies have demonstrated that, following intervention with OGA inhibitors in model mice, RNA sequencing analysis revealed that OGA inhibitors can enhance O-GlcNAc expression and upregulate the expression of genes related to learning, cognition, and behavior (Bell et al., 2023).

## 4 Glycosyltransferase

Glycosyltransferases are enzymes that catalyze the transfer of sugar groups from active sugar donor molecules to receptor molecules (Lairson et al., 2008). Significant differences in glycosyltransferases within the N-glycan biosynthesis pathway have been identified in the cerebrospinal fluid and autopsy samples of patients with SCZ (Stanta et al., 2010). These enzymes facilitate the proper folding of newly synthesized proteins through N-glycosylation modifications in the ER and ensure that misfolded proteins are recognized and degraded through quality control mechanisms such as the calreticulin and calnexin cycles (Molinari et al., 2005; Tannous et al., 2015). SCZ has been shown to involve abnormalities in multiple neurochemical systems, with the core issue potentially being a disorder of the intracellular ER quality control system (Patel et al., 2017; Kim et al., 2021). In the DLPFC of patients with SCZ, the level of the ER processing-related glycoprotein the UDP-Glc: Glycoprotein Glucosyltransferase (UGGT) 2 is significantly increased (Kim et al., 2018). UGGT family of enzymes is responsible for the terminal Glc modification in the N-glycosylation of proteins, ensuring the correct folding of glycoproteins in the ER and participating in protein quality control (Ito et al., 2015). During glycosylation, if a protein is correctly folded, the Glc is completely removed, allowing the protein to be transported to the Golgi apparatus for further processing. If the protein is not correctly folded, UGGT recognizes this misfolded state and reattaches a Glc molecule to the oligosaccharide chain, returning the glycoprotein to the calreticulin/calnexin cycle for further folding (Parodi, 2000). The increased expression of UGGT2 suggests that the ER quality control system may be disrupted in patients with SCZ, leading to repeated processing and verification of proteins and affecting the glycosylation process.

Additionally, Kippe et al. (2015) observed the downregulation of *β*1,3-N-acetylglucosaminyltransferase-8 (*β*3GnT8) and Mannosyl (*α*-1,3-)-Glycoprotein *β*-1,4-N-Acetylglucosaminyltransferase, Isozyme A (MGAT4A) in DLPFC samples from elderly autopsies. B3GNT8 and MGAT4A are crucial members of the GlcNAc transferase family and play pivotal roles in the synthesis and modification of N-glycans. Specifically, *β*3GnT8 catalyzes the formation of *β*1-6 branched N-glycans on lactosamine, which subsequently modifies cell surface glycoproteins. This enzymatic activity regulates cell communication and recognition and influences the formation and functionality of the extracellular matrix in nervous system cells (Liu et al., 2014). MGAT4A is involved in protein quality control through the modification of N-glycans, ensuring proper protein folding and function, and playing a role in cell signaling and immune responses (Krishnan and Krishnan, 2021). Downregulation of B3GNT8 and MGAT4A in DLPFC tissues may result in abnormal glycan structures and functions, ultimately interfering with neuronal function and cognition.

A genetic study involving 108 SCZ-related loci revealed that genes associated with cognitive function were significantly enriched in the Fuc metabolism pathway, suggesting a close relationship between cognitive function in SCZ patients and abnormal Fuc metabolism (Fabbri and Serretti, 2017). Fuc is a six-carbon sugar with the chemical formula C6H12O5. Its molecular structure differs from that of other common monosaccharides, lacking a hydroxyl (-OH) group, and is therefore also known as a deoxy sugar (Schneider et al., 2017). Muller et al. found that the protein expression of O-fucosyltransferase 2 (POFUT2) and FUT8 in the STG of SCZ patients was significantly altered (Mueller et al., 2017). Specifically, the protein expression of POFUT2 increased, while the protein expression of FUT8 decreased. The reduced expression of FUT8 led to a decrease in the *α*-1,6-fucosylated N-glycoprotein, the reaction product of FUT8, indicating that the reduced expression of FUT8 has practical functional consequences. POFUT2 is a glycosyltransferase that transfers Fuc from the donor molecule GDP-Fuc to specific protein substrates to form O-fucosylation modifications (Sanz-Martínez et al., 2022). Since POFUT2 can recognize and modify the formation of disulfide bonds, stabilize the TSR domain of fucosylation, and ensure the folding and transport of proteins in the ER pathway, an increase in its expression level may lead to excessive O-fucosylation modifications (Takeuchi et al., 2017). This excessive modification could subsequently alter the dynamic folding process of proteins and the ER quality control system, potentially resulting in excessive protein folding or abnormal passage through the quality control system. When these proteins accumulate in the extracellular matrix, they further affect neuron signaling and the stability of the extracellular environment. Additionally, a genetic study found that the AMAMTS9 gene, a key substrate of POFUT2, has a devastating effect on structural stability in Indian families with SCZ (Wang et al., 2007; Shekhar et al., 2023). Its gene variants may disrupt the glycosylation process, affecting the formation of the extracellular matrix and the synaptic function of neurons, thereby influencing the pathophysiology of SCZ. FUT8 is a key *α*-1,6-fucosyltransferase responsible for the core fucosylation of N-glycoproteins (García-García et al., 2021). Animal studies have shown that FUT8 knockout mice not only exhibit a schizophrenic phenotype and reduced working memory but also have a significant increase in the post-synaptic density of AMPA receptors in the hippocampus (Gu et al., 2015). Treatment with haloperidol restored their motor and social abilities to wild-type mouse levels. Lu et al. (2019) further found that the SCZ-like behavior of FUT8 knockout mice was accompanied by increased activation of microglia and astrocytes and persistent neuroinflammation. Moreover, FUT8 deficiency not only increases the sensitivity of cells to pro-inflammatory factors but also inhibits the transmission of anti-inflammatory signals, such as TGF-*β* (Venkatachalam and Weinberg, 2013).

## 5 Polysialic acid - Neural cell adhesion molecule (PSA-NCAM) in SCZ

Sia is an acidic monosaccharide with nine carbon atoms, typically in the form of N-acetylneuraminic acid (Schauer and Kamerling, 2018). PSA is a chain of multiple Sia residues linked by *α*2,8-glycosidic bonds. The synthesis of PSA primarily occurs in the cytoplasm and Golgi apparatus, beginning with GlcNAc and phosphoenolpyruvate and culminating in the addition of Sia residues to the ends of oligosaccharide chains on glycoproteins or glycolipids (Sato and Kitajima, 2021). The resultant PSA chains can significantly extend the length of sugar chains, thereby altering the structure and function of the associated proteins or lipids (Bu et al., 2023). NCAM is the most extensively studied transmembrane glycoprotein involved in polysialylation and belongs to the immunoglobulin superfamily. NCAM comprises five immunoglobulin domains, two fibronectin III domains, and both transmembrane and intracellular domains (Rutishauser et al., 1988; Weledji and Assob, 2014). The polysialylation of NCAM primarily occurs on its immunoglobulin-like domains, where Sia molecules are synthesized into PSAs and attached to NCAM by two sialyltransferases, ST8 *α*-N-acetyl-neuraminide *α*-2,8-sialyltransferase 2 (ST8SIA2) and ST8 *α*-N-acetyl-neuraminide *α*-2,8-sialyltransferase 4 (ST8SIA4) (Mori et al., 2017). Furthermore, the expression and activity of ST8SIA2 and ST8SIA4 at various developmental stages dictate the function and distribution of PSA-NCAM. Studies have demonstrated that PSA-NCAM facilitates neuronal migration and positioning during development by decreasing cell surface adhesion and increasing intercellular repulsion (Guan et al., 2015). Additionally, it promotes synaptic dynamics and remodeling by reducing neuronal adhesion through its high negative charge and hydrophobicity, thereby enhancing learning and memory (Vukojevic et al., 2020). Moreover, PSA-NCAM can mitigate immune cell attacks on neurons and shield them from immune system-induced damage (Gretenkort et al., 2023). Regarding synaptic formation and stability, elevated levels of polysialylation can also enhance axon growth and regeneration (Chattopadhyaya et al., 2013). Consequently, PSA-NCAM influences neurodevelopment, neuronal migration, synaptic formation, and neural plasticity.

A previous study indicated a reduction in the number of PSA-NCAM immune response cells in the brains of patients with SCZ, with no significant changes in NCAM observed in Western blotting experiments (Barbeau et al., 1995). This finding suggests that the decrease in PSA-NCAM immune response cells may be closely related to PTMs of the protein. Subsequently, Vawter et al. deglycosylated NCAM180 to low molecular weight cN-CAM (deglycosylated to the basic glycan structure) and dN-CAM (deglycosylated to very few glycan structures) using neuraminidase. They found that cN-CAM content in the hippocampus of patients with SCZ was increased, accompanied by a decrease in multiple sialylations of NCAM180 (Vawter et al., 2001). This suggests that NCAM180 in the hippocampus of patients with SCZ may be more susceptible to proteolysis, and that its loss of polysialylation affects the regulation of synaptic adhesion. A recent correlation analysis showed significantly elevated PSA levels in the serum of patients with SCZ, which correlated with decreased hippocampal gray matter volume (Müller-Miny et al., 2023). Elevated PSA levels reflect a reduction in the sialylation process, leading to insufficient sialylation of the neuronal surface. This makes neurons more susceptible to attack and removal by the complement system and microglia, resulting in a decrease in the number of neurons and hippocampal gray matter volume (Allendorf et al., 2020). Furthermore, neuroimaging studies have found that increased PSA-NCAM levels in patients with SCZ are associated with reduced brain integrity compared to healthy controls and are significantly positively correlated with the PANSS negative scale (An et al., 2020).

Due to the significant role of NCAM’s PSA-dependent modification in neurodevelopment, several genetic studies have identified ST8SIA2 as a key candidate gene for SCZ. This finding has been corroborated in SCZ patients from Australia, Korea and northeastern Italy (Vazza et al., 2007; McAuley et al., 2012; Yang et al., 2015). Within the PSA transferase family, ST8SIA2 is primarily expressed during early developmental stages, whereas ST8SIA4 is active throughout development (Röckle and Hildebrandt, 2016). Research has shown that ST8SIA2 and ST8SIA4 double knockout mice completely lack PSA levels, leading to the gradual disappearance of the thalamic reticular nucleus after birth (Kröcher et al., 2015). This results in guidance defects and incorrect neural pathways in the mice. Further studies have found that ST8SIA2 knockout mice exhibit severe memory deficits, a lack of pleasure-seeking behavior, and increased sensitivity to amphetamine (Kröcher et al., 2015). These symptoms are accompanied by thalamocortical connectivity defects, reduced internal capsule volume, and dilated ventricles. These defects were not observed in ST8SIA4 knockout mice, indicating that the PSA synthesis defect caused by ST8SIA2 deficiency affects early neurodevelopment and induces the emergence of SCZ-like behaviors (Kröcher et al., 2015). In a large Australian cohort study, diffusion-weighted imaging measurements showed that the protective haplotype of the ST8SIA2 gene was associated with improved white matter microstructure (increased anisotropy scores) in healthy controls (Fullerton et al., 2018). However, this protective effect was reversed in patients with SCZ, who exhibited decreased anisotropy scores and IQ. This further reveals the complex mechanism of ST8SIA2 in SCZ. Additionally, another study revealed that, compared to healthy controls, there were no significant differences in the expression levels of NCAM, PSA-NCAM, ST8SIA3, and ST8SIA4 in the STG of SCZ patients. However, the protein expression of ST8SIA2 was significantly elevated (Mueller T. M. et al., 2018). This elevation may impact the synthesis of PSA-NCAM, suggesting that impaired glycosyltransferase function in the STG of SCZ patients may trigger a compensatory mechanism involving ST8SIA2 upregulation.

## 6 The effect of antipsychotic drugs on SCZ glycosylation

Antipsychotic drugs have significantly advanced in alleviating the positive symptoms of SCZ, particularly by regulating the dopamine and serotonin systems (Stępnicki et al., 2018). However, these drugs still face substantial limitations in managing side effects and improving negative and cognitive symptoms. As previously mentioned, abnormal glycosylation modifications play a crucial role in receptor function, synaptic plasticity, and neuronal function in patients with SCZ. Targeting these modifications could potentially improve positive, negative, and cognitive symptoms concurrently. Notably, due to the long half-life of antipsychotic drugs in autopsy samples, some researchers have analyzed these samples to investigate the effects of antipsychotic drugs on glycosylation modifications (Alnafisah et al., 2022). A study focusing on the entire course of SCZ disease development revealed significant enrichment in the glycosylation of biopolymers and amino acid glycosylation pathways in postmortem DLPFC samples from patients treated with chlorpromazine for an intermediate period (12–18 years) (Narayan et al., 2008). A non-targeted proteomics study of adipose tissue from rats with a methylazoxymethanol acetate-induced SCZ-like model demonstrated significant changes in N-glycosylation levels of proteins following intervention with olanzapine, risperidone, and haloperidol (Kucera et al., 2021). Another study analyzing the serum proteome of patients with acute paranoid SCZ using high-throughput hydrophilic interaction chromatography found that olanzapine-induced changes in serum glycoproteins were primarily characterized by alterations in galactosylation and sialylation levels, without affecting the branching structure of the glycan (Telford et al., 2012). These changes were not observed in the low-abundance serum proteome, indicating that olanzapine alters the glycosylation pattern but did not fully restore normal glycosylation levels. In contrast, another study found that olanzapine also inhibits the expression of the B4GALT1 gene, which encodes Gal-transferase activity, and alters the expression of other glycosylation-processing enzymes in the hepatic tissues of SCZ patients (Su et al., 2020). Furthermore, a decrease in B4GALT1 levels was observed in the STG tissue of elderly SCZ patients receiving treatment with atypical antipsychotic drugs (Mueller T. M. et al., 2019). It is important to note that although existing studies have revealed the multifaceted effects of antipsychotic drugs on glycosylation, most of these studies rely on postmortem samples, which are limited by quality, timing of collection, and accuracy of the patient’s medication history. Although postmortem samples can provide preliminary evidence of the long-term effects of drugs on glycosylation, they are insufficient to reflect the dynamic changes in glycosylation during life.

## 7 Conclusions and outlook

As a complex and precise PTM, glycosylation plays multiple key roles in both health and disease states, providing a solid scientific foundation for precision medicine and the development of novel treatment strategies. This review summarizes the existing literature, detailing N-glycosylation, O-glycosylation, glycosyltransferases, PSA-NCAM, and their potential mechanisms in SCZ. It analyzes the dynamic changes of glycosylation in different brain regions and cellular environments (particularly protein folding and transport) and summarizes the regulatory effects of antipsychotic drugs on glycosylation in SCZ patients. Although existing studies have highlighted the importance of glycosylation in the pathophysiology of SCZ, they have primarily focused on phenotypic changes in glycosylation and specific glycosylation modifications. These studies often use singular methods, lacking a comprehensive and systematic analysis of the entire glycosylation modification network. Further research using multiple high-sensitivity and high-resolution techniques (such as mass spectrometry and glycomics) is needed to fully elucidate the complete picture of glycosylation modifications. Future research should focus on elucidating how glycosylation affects the structure, function, and localization of proteins in neurons, thereby revealing its specific role in the pathogenesis of SCZ. Additionally, the expression of glycosylation modifications at different developmental stages and in various brain regions exhibits clear temporal and spatial specificity, with contrasting pathological characteristics. Combining single-cell sequencing technology and high-resolution imaging techniques, further studies should explore the dynamic changes in glycosylation modifications across different stages of SCZ and brain regions.

In summary, although the study of glycosylation in SCZ faces many challenges, it also holds great potential. By thoroughly analyzing the mechanisms of glycosylation modification, exploring its specific role in SCZ, and developing new diagnostic markers and treatment strategies, it is hoped that breakthroughs will be made in understanding the etiology and improving the clinical treatment of SCZ. Despite the limited number of existing studies, with technological advancements and deepening research, the study of glycosylation will undoubtedly open new prospects for the prevention and treatment of SCZ.
